# A novel method for ZnO@NiO core–shell nanoparticle synthesis using pulse laser ablation in liquid and plasma jet techniques

**DOI:** 10.1038/s41598-023-32330-z

**Published:** 2023-04-03

**Authors:** Hadeel J. Imran, Kadhim A. Hubeatir, Kadhim A. Aadim

**Affiliations:** 1 Laser and Optoelectronics Engineering Department, University of Technology-Iraq, Baghdad, Iraq; 2 Department of Physics, College of Science, University of Baghdad, Baghdad, Iraq

**Keywords:** Biotechnology, Medical research, Engineering, Materials science, Nanoscience and technology, Physics

## Abstract

Given their versatile nature and wide range of possible applications, core–shell nanoparticles (NPs) have received considerable attention. This paper proposes a novel method for synthesizing ZnO@NiO core–shell nanoparticles using a hybrid technique. The characterization demonstrates the successful formation of ZnO@NiO core–shell nanoparticles, which have an average crystal size of 13.059 nm. The results indicate that the prepared NPs have excellent antibacterial activity against both Gram-negative and Gram-positive bacteria. This behavior is primarily caused by the accumulation of ZnO@NiO NPs on the bacteria's surface, which results in cytotoxic bacteria and a relatively increased ZnO, resulting in cell death. Moreover, the use of a ZnO@NiO core–shell material will prevent the bacteria from nourishing themselves in the culture medium, among many other reasons. Finally, the PLAL is an easily scalable, cost-effective, and environmentally friendly method for the synthesis of NPs, and the prepared core–shell NPs could be used in other biological applications such as drug delivery, cancer treatment, and further biomedical functionalization.

## Introduction

Nanoparticles are currently considered a powerful tool and the most effective area for research studies due to their unique properties that depend on their size. The prefix "Nano" stands for ten powers to minus nine powers, which is called a nanometer-scale^1^. Particles with a diameter of under 100 nm are known as nanoparticles. Metal nanoparticles (NPs) have significant benefits in a variety of fields, including medicine, biosensing, biomedical sciences, cosmetics, food, and electronics^2,3^.

Scientists and researchers have a great deal of interest in the hybridization of various elements at the nanoscale because of their unique physicochemical features, such as electrical, optical, catalytic, and thermal^4^. These unique and novel properties result from combining the characteristics of various materials and the effect of particle size reduction from macro to nanostructure, which leads to an increase in the surface-to-volume ratio, followed by a complete change in the physicochemical properties^5–7^.

In recent years, a new type of hybrid NPs called "core–shell NPs" has been developed, consisting of two or more types of single nanomaterials^8^. Researchers have found that the majority of NPs' physical characteristics are dependent on their nanostructured surfaces due to the characteristics of NPs that help increase the number of dangling bonds that affect their physicochemical properties. These qualities could be enhanced still further by using a coating material to create the outer shell of this nanostructured form through chemical passivation as a subsequent reduction process of the core. This process is known as a "core–shell" formation. Also, the shell layer could improve the physiochemical properties of the core material, like its catalytic activity and nonlinear properties^9^, leading to new, unique properties that could speed up development in several application fields^10,11^.

Research interest in zinc oxide (ZnO) has been increasing, particularly in nanotechnology, to synthesize ZnO on a nanoscale because of its properties and applications^12–15^. It is crucial in the creation of possible antimicrobial drugs as well as in scientific and technological fields such as nonlinear optics, electrical devices, catalysis, and medicinal applications for their having a large surface area and high crystallinity^16,17^. ZnO NPs have been employed as antibacterial agents due to their effectiveness against pathogen-resistant strains, low toxicity, and heat resistance^18–20^. The performance of the metal oxide semiconductor’s photocatalytic system is enhanced by using transition metals such as Fe, Co, Ni, and Mn. Because nickel has the same valence state and ionic radius as other transition metals, it can be added to them (as core@shell) to increase their photocatalytic and antibacterial activity. In addition, it exhibits excellent photocatalytic and antibacterial activities when used as a core with different metal oxides^21–24^. In this work, we chose NiO to be the shell for ZnO because NiO has the same characteristic not found in other materials; the most important one is the transition through the cell, which gives him the ability to hold the core material to the cells. This property enhances the effect of the core material and opens the door to a huge number of applications, especially in the biomedical field and drugs. This structure is first proposed in this work.

There are numerous techniques for synthesizing the nanoparticles, including pulsed laser deposition, sol–gel preparation, chemical coprecipitation, thermal decomposition, hydrothermal methods, etc.^8,25,26^. Recently, a hybrid method has been used that includes the pulse laser ablation in liquid (PLAL) and the plasma jet technique because of their many advantages, including cost-effectiveness, environmental friendliness, and a lack of need for expensive equipment^27,28^. Also, they don’t need a loge time in preparation just a few mints. The primary mechanism for PLAL nanomaterial fabrication can be illustrated as follows: laser-matter interaction (absorption of a laser pulse by a material); the formation, expansion, and cooling of a plasma plume; the generation of shock waves; and the formation, expansion, and collapse of bubbles in a liquid medium^14–17^. While in the plasma jet technique a wide range of reactive oxygen and nitrogen species (RONS) produced by atmospheric pressure non-thermal plasma sources is crucial to this application^22,29^. Since plasma-generated RONS alter the chemical makeup of a liquid, plasma-liquid interactions are crucial in the production of nanomaterials^29–33^. Ozone, singlet oxygen, hydrogen peroxide, hydroxyl radicals, and nitric oxide are just a few of the common RONS that are created during plasma-liquid interactions^8,34^.

Alcohol and betadine (Povidone Iodine) are potent microbicides that can eliminate all kinds of bacteria, viruses, fungi, and other microorganisms^35^. According to Lavelle et al., some patients’ iodine absorption caused unexplained abnormalities and renal failure^36^. This solution is safe to use on dried skin, but if applied to a wound, it may cause allergic reactions in some users and cause severe irritation (Hwang et al., 1986), and it will also delay the healing process, leaving scars behind^37^. Furthermore, it shouldn't be applied to surgical incisions or wounds like burns, and iodine is absorbed by pregnant women, harming their unborn children. However, the issue with antibiotics is the emergence of resistant strains and the potential for microbial strains to become resistant to one or more antibiotics. If the feeding tissue becomes infected, resistant wounds spread quickly and may even result in the patient's death. When severe crises like earthquakes, floods, or large fires suddenly result in a large number of patients inflicted with superficial and deep wounds in hospitals, controlling persistent strains and preventing epidemics has become especially challenging^38^.

Researchers are now focusing their attention on alternative, newer antimicrobial agents to replace common antimicrobials or to use them occasionally to prevent the epidemic of resistant strains due to increased community awareness of health, the apparent drawbacks of betadine and alcohol, and the development of multi-resistant microbes to several drugs^19,39^. Nanotechnology is a multidisciplinary field that combines basic and applied sciences such as biophysics, molecular biology, and bioengineering^40^. Size reduction is a fundamental unit operation that has a wide range of applications in pharmacy^41,42^.

To the best of our knowledge, there are no studies synthesizing the ZnO@NiO (ZNO) core–shell NPs structure. In addition, we propose a new hybrid method for synthesis of the core–shell structure that includes two-step PLAL and plasma jet.The primary goal of this research is to synthesize ZNO NPs, use them as antibacterials, and determine their effect on cells (white blood cells).This is also the first study of the effect of ZNO NPs on *Escherichia coli* (Gram-negative) and *Staphylococcus aureus* (Gram-positive). Also, the fluorescence microscopic image was used to show the bacteria strain before and after treatment. The morphological and optical properties of the prepared NPs are investigated using TEM, FESM, AAS, EDX, XRD, and UV–Vis.

## Result and discussion

### Hybrid method

**Figure 1:**
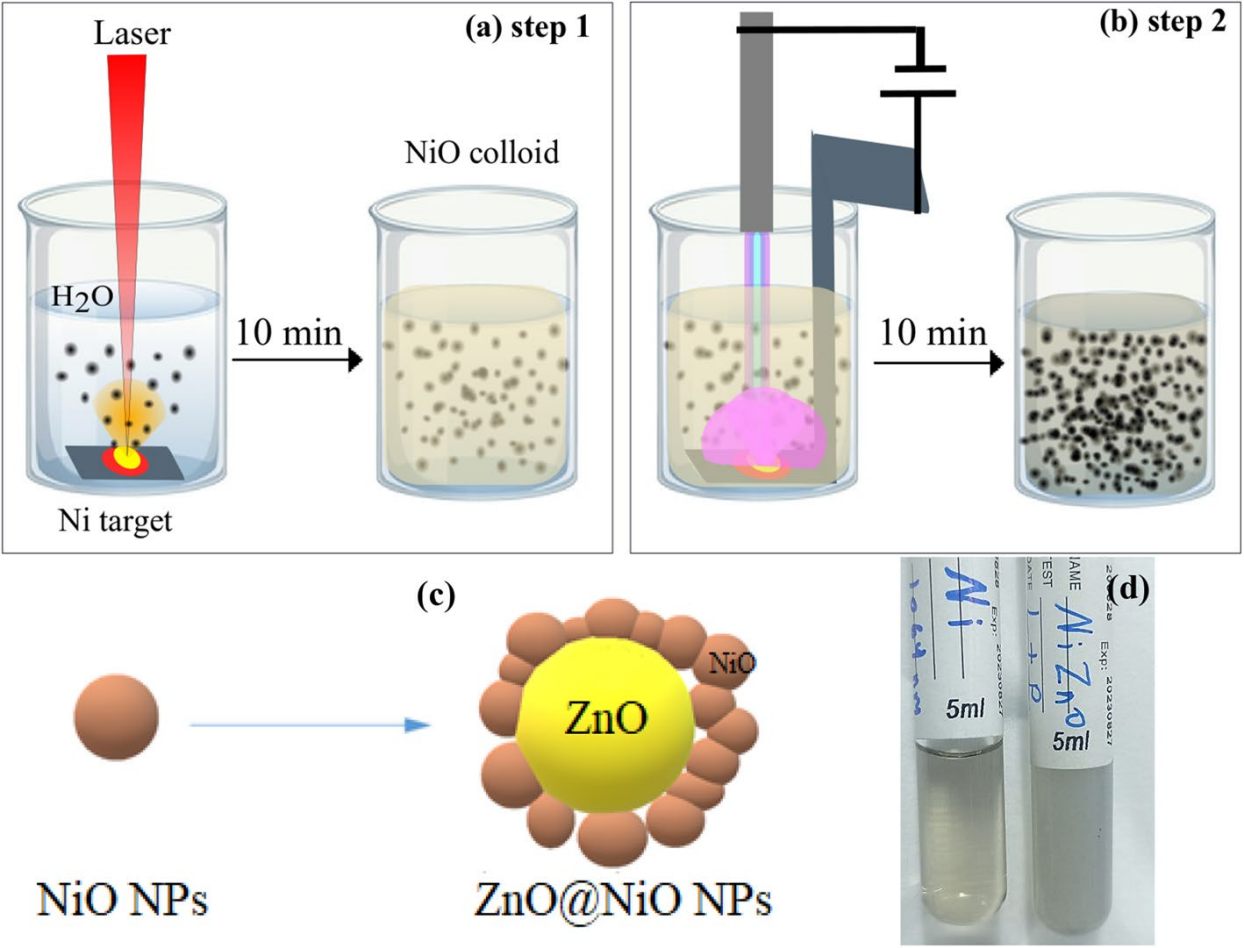
Illustration of the hyprid approach to preparing NiO and ZNO NPs colloids: (**a**) NiO colloids by PLAL, (**b**) ZNO NPs colloids usinf plasma jet, (**c**) scheme configuration of core–shell particles, (**d**) Photographs for the prepared colloids of NiO and ZNO NPs.

A novel technique has been used to prepare the ZNO core–shell NPs called hybrid techniques, which includes two steps. First, the Ni metallic pellet was immersed in distal water and ablated with the Nd:YAG laser at 800 mJ for 10 min. Second, the Ni pellet was replaced with a Zn pellet and ablated using a plasma jet with 12 kV and 3 m^3^/s for 10 min in the same medium (NiO NPs colloidal). The hybrid techniques successfully formed ZnO@NiO (ZNO) core–shell NPs, which showed evident color-changing (Fig. 1). The NiO NPs were synthesized first in distal water, and the water color changed to light yellow transparent colloidal (can see-through it) and then converted to gray opaque or non-transparent colloidal (can not see through it) after the formation of the ZNO core–shell (Fig. 1d). The concentration of each particle was measured using AAS. In this process, ZnO is the core and NiO is the shell. The formation process is explained as follows: The laser ablation cycle in water starts with the laser beam on the target surface submerged in a liquid medium. Plasma is generated immediately by the laser beam, which is called "laser-induced plasma," removing the surface of the solid target. After plasma development in the liquid atmosphere, the plasma is immediately confined by the liquid medium; hence, the thermodynamic state of the laser-induced plasma is entirely different^18,43^. Then the ablated atoms from the target get aggregated in the water and make the nanoparticles. In the plasma jet case, the generated plasma ionizes the colloidal solution's water and NiO NPs, causing an expansion in the Zn surface layer and allowing the small ion to easily accommodate between the layers of Zn.This causes the lattice space to expand, greatly aiding the cleavage of layered materials upon rapid heating. So that the bulk layered materials exfoliate into smaller layers or nano-size platelets as a result of the intercalant's dramatic expansion caused by the heat. These is the ZnO NPs which immediately surrounded by the NiO ions and made a ZNO core–shell NPs.

### Morphological characterization of ZNO core/shell nanoparticle

**Figure 2:**
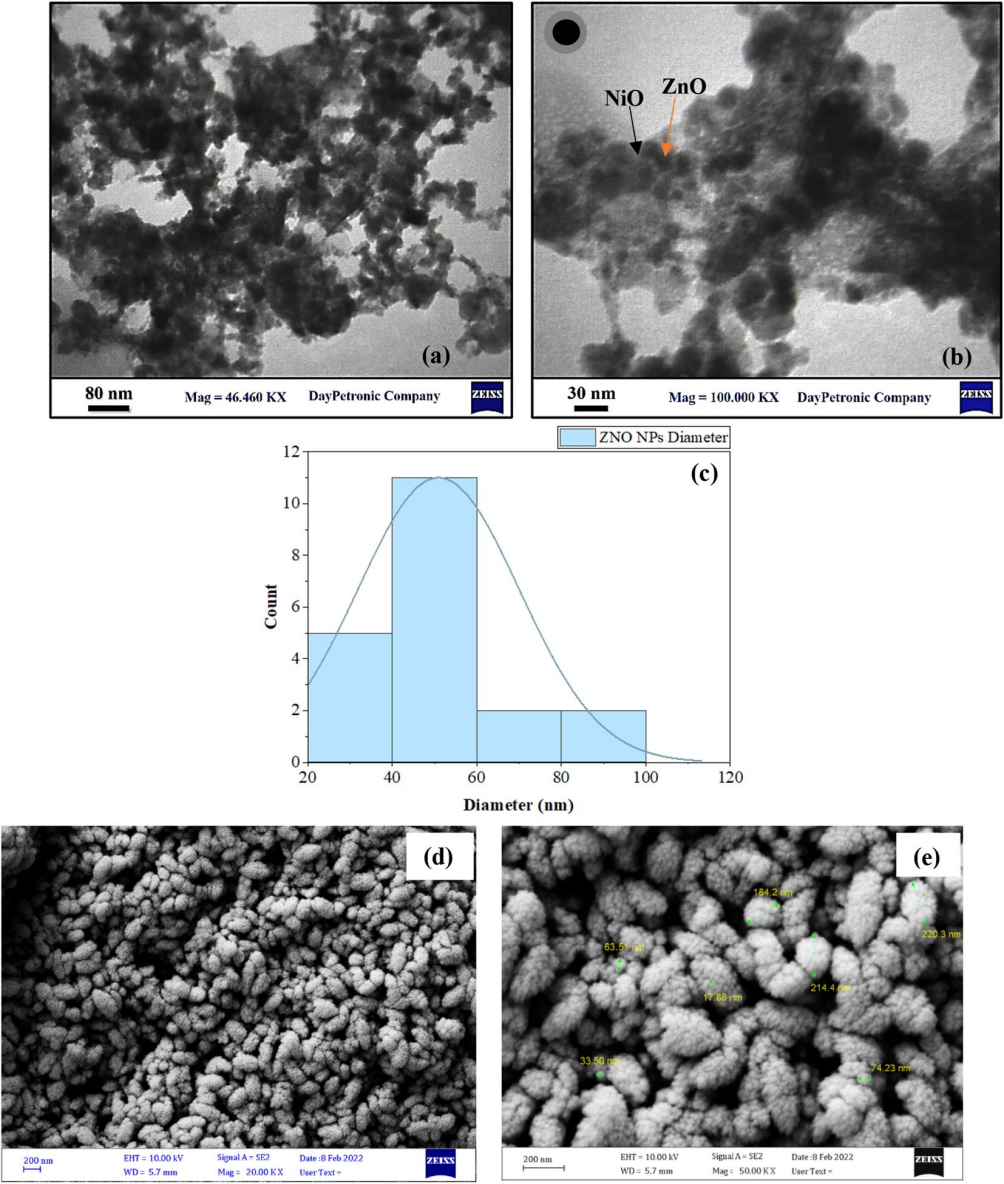
(**a**) TEM image of the prepared ZNO NPs at 80 nm, (**b**) TEM image of the prepared ZNO NPs at 30 nm, (**c**) histogram of ZNO NPs, (**d**) EDX spectrum of ZNO NPs, (**e**) and (**f**) FESEM image of ZNO NPs at 200 nm.

**Figure 3:**
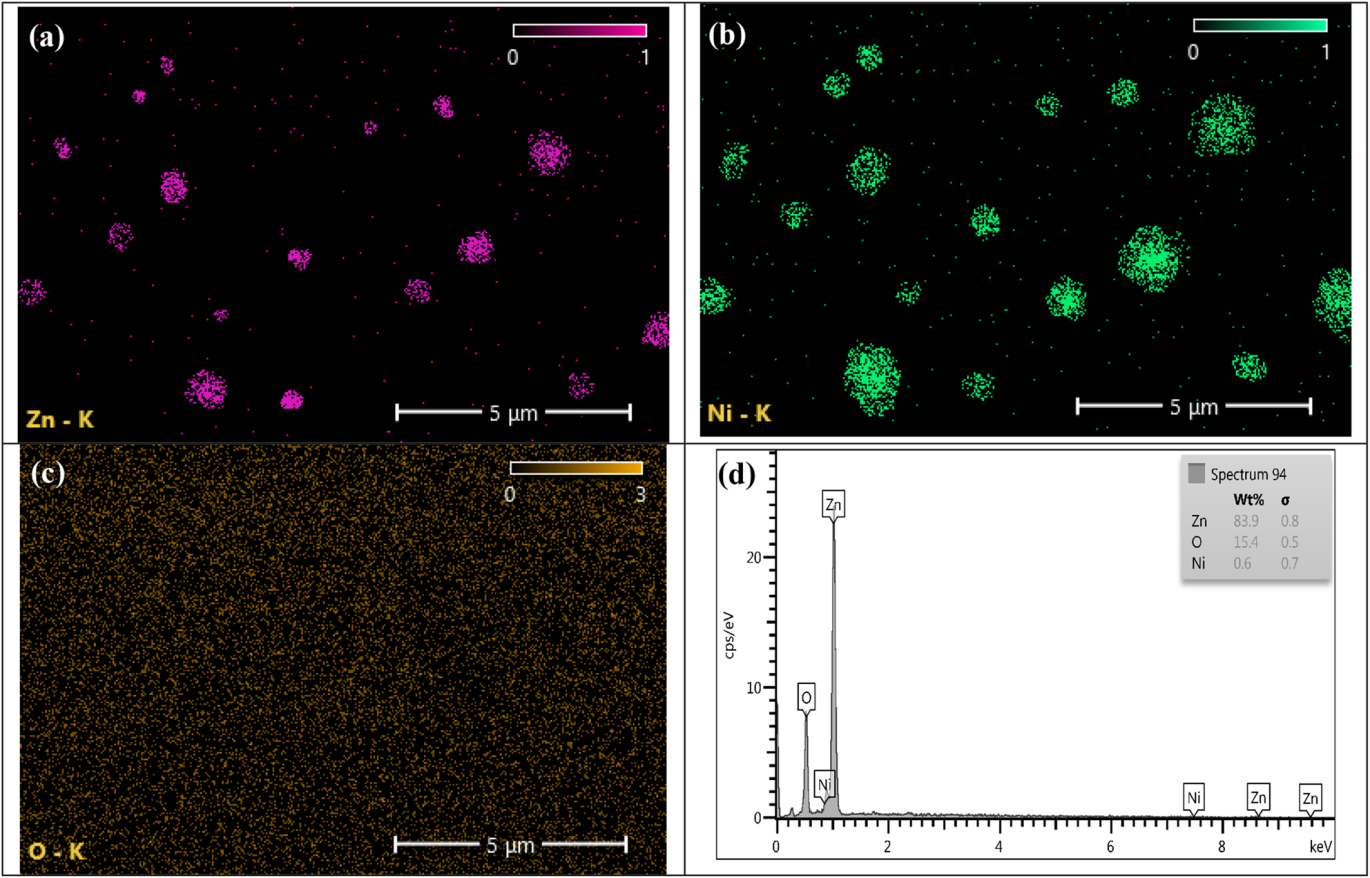
The EDX mapping and EDX spectrum of ZNO NPs, (**a**) EDX mapping of Zn, (**b**) EDX mapping of Ni, (**c**) EDX mapping of O, and (**d**) EDX spectrum of ZNONPs.

The transmission electron microscope (TEM) was used to show the structure of the prepared ZNO NPs. Which confirm the formation of the core–shell structure, where, as shown in Fig. 2a and b, the dark point is the ZnO NPs (core) and is surrounded by the light point, which represents the NiO NPs (shell). Also, the TEM was taken for NiO alone before adding the ZnO and the image (shown in Fig. S3, which confirms that the light spot is the NiO NPs. From these images, it was clear that very small particles are formed, ranging in size from 20 to 100 nm, with an average particle size of 50.9 nm and a nearly spherical shape (shown in Fig. 2c). The NiO particle shown was so small because it was ionized, and the cluster formed in the PLAL part is broken due to the effect of the plasma jet technique. The FESEM image shown in Fig. 2e and f shows the morphology and distribution of the core–shell NPs formed. It showed a uniform distribution and has an elliptical shape. It also shows how much the NiO NPs cover the ZnO NPs and how much of that particle is small. The EDX mapping ad spectrum result shows the successful formation of ZNO core–shell NPs as shown in Fig. 3. Where Fig. 3a and b reveals the aglomaration of znic and nickal anoparticle formation and Fig. 3c show the uniform distribution of oxygen they are indicted with rosy, green, and yallow respectively, which suggests the successful encapsulation of ZnO with NiO. As it was shown in Fig. 3d, there is a very small amount of NiO in the colloidal solution, which confirms that it was converted to a shell for ZnO. This result of ZnO was ageed with S. Sardar et al. result where also show the formation of spherical shape^44^ This result also agreed with the AAS result, which gives 50 ppm for ZnO and 5.8 ppm for NiO, while when we measure the NiO after PLAL, it gives 60 ppm, and this is also another indication that all the ZnO particles are covered with NiO particles because it has a higher concentration in the used colloidal.

**Figure 4:**
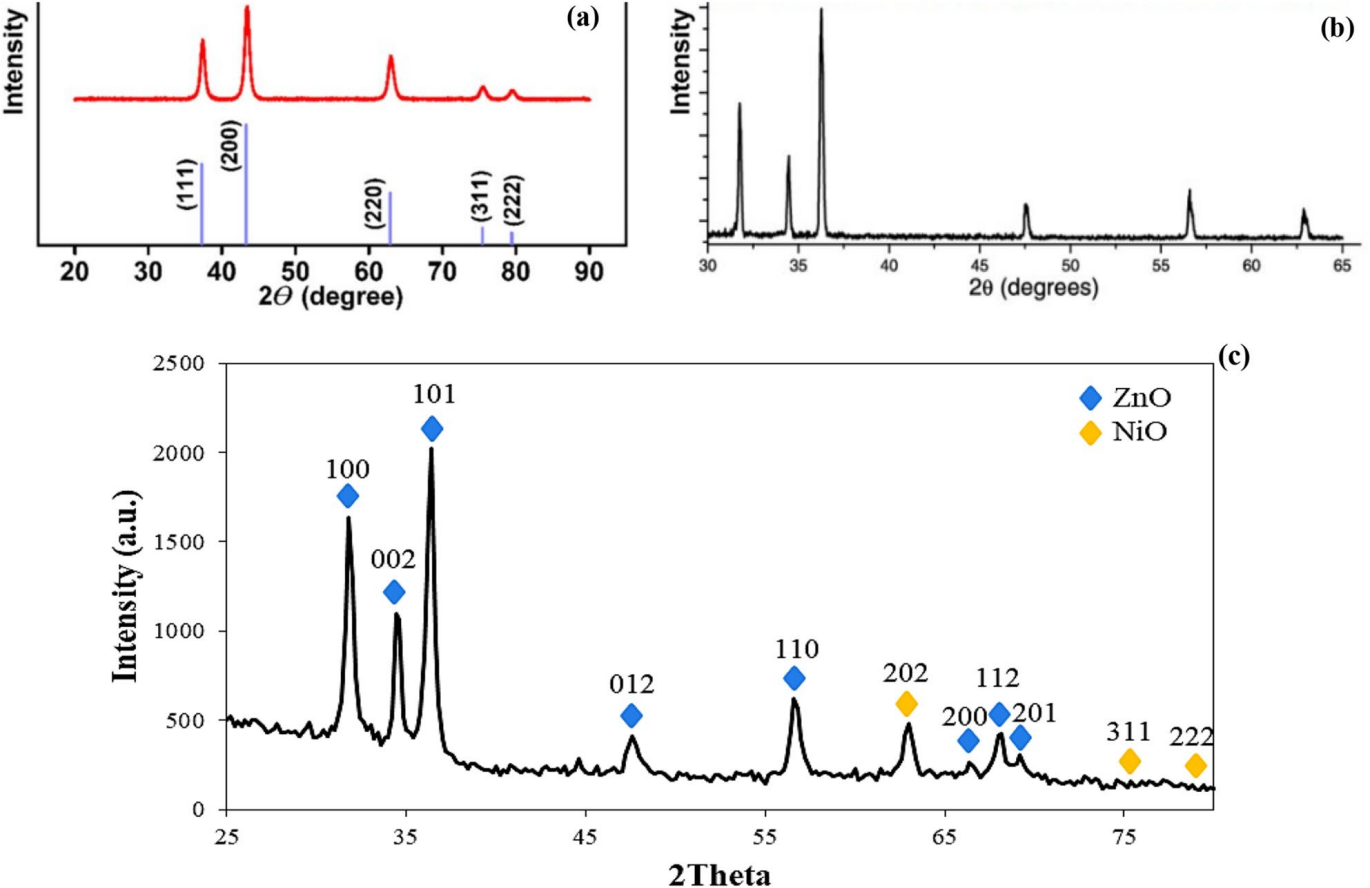
XRD pattern of the prepared sample, (**a**) XRD of ZnO, (**b**) XRD of NiO, (**c**) XRD of the synthesized ZNO core/shell NPs.

Figure 4 shows the XRD pattern for ZNO core–shell NPs and the standard XRD data for both ZnO and NiO^6^. Diffraction peaks were observed at 2θ = 31.856°, 34.52°, 36.346°,47.616°, 56.652°, 66.412°, 68.035°, and 69.221° which were indexed to Miller indices of (100), (002), (101), (012), (110), (200), (112), and (201) respectively. The highest diffraction pattern between 30 and 40° indicates the formation of wurtzite ZnO, which was consistent with JCPDS 96–900-4181. The diffraction pattern indicates the formation of NiO with an observed peak of 2θ = 62.945°, 75.435°, and 79.621°, corresponding to (202), (311), and (222) planes, respectively according to JCPDS 96-432-9326. It also indicates cubic phase crystallites of NiO, which is consistent with the first step agree with M.Patel et al.^45^. The average crystal size was determined using Debye–Scherrer Eq. (1), where the ZnO NPs are 13.81 nm, and the NiO is 11.08 nm and 13.059 for ZNO NPs (see Table S1, the XRD peaks details). These results indicate the successful formation of nanometer-sized particles which agreed with the FESEM result. This result agreed well with Cheng et al., Gnanamozhi et al. and Xie et al.^21,46,47^. The differeaction pateren in Fig. 3c ac compared with Fig. 3a and b (the stander data) it show an agreement with both ZnO peak and NiO peak which confirm the formation of the core/shell without any chemical reaction between them.

### Optical properties

**Figure 5:**
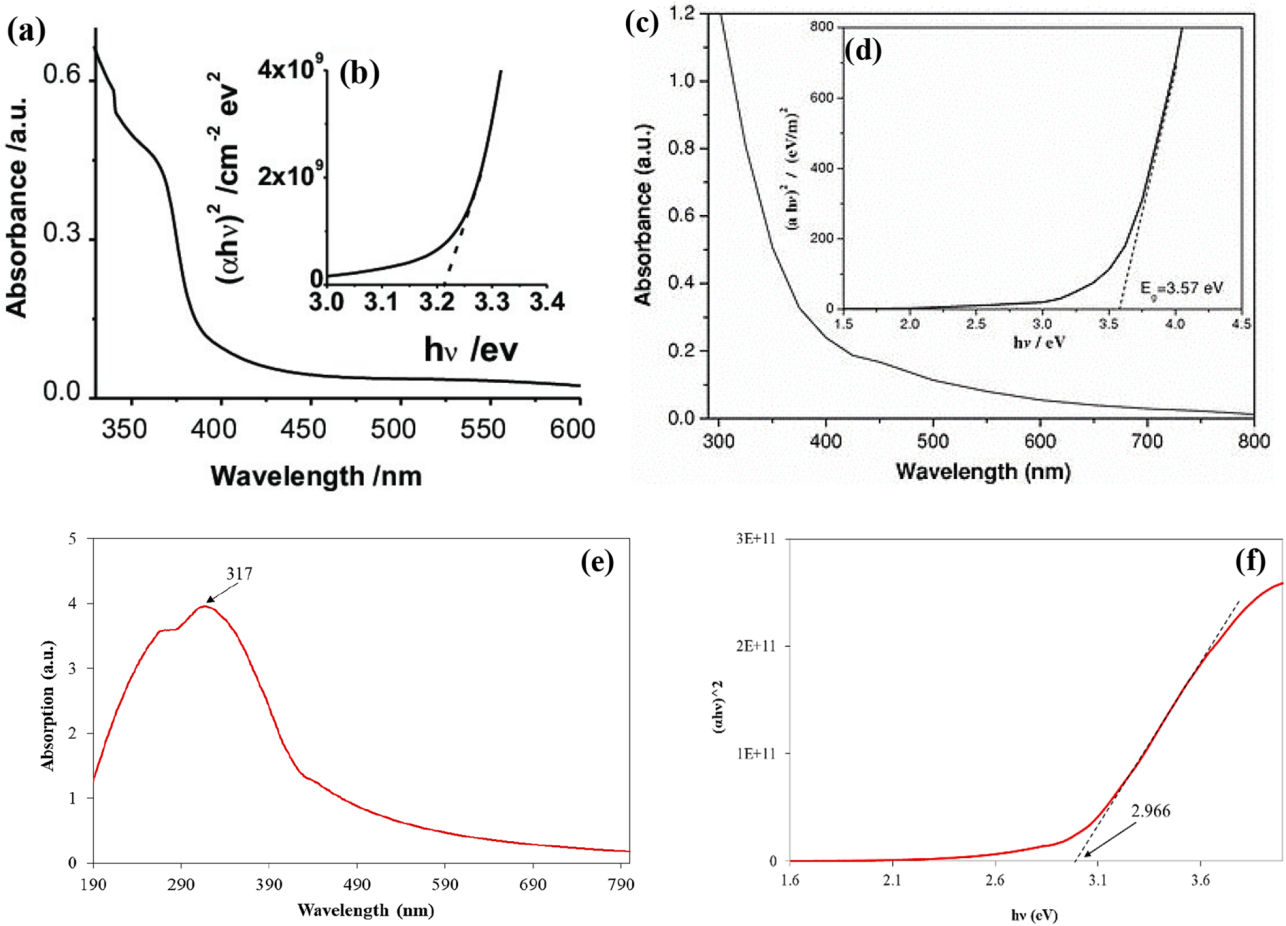
The optical properites of the NPs (**a**) Optical absorption of ZnO, (**b**) the band gap of ZnO, (**c**) Optical absorption of NiO, (**d**) the band gap of NiO, (**e**) th optical absorption of the prepared ZNO NPs, (**b**) Optical bandgap energy of ZNO NPs.

The optical absorption of the ZNO core–shell NPs was measured using a UV–vis spectrophotometer, providing information on the physical properties of the prepared NPs such as the absorption and energy band gap. Figure 5a and b show the absorption and bandgap of ZnO and NiO respectively to compare them with prepared core/shell^48,49^. Figure 5c shows the absorption of ZNO core–shell NPs. The spectra show that the small shift in the edges where light is absorbed is caused by the addition of Ni. When compared to the spectra of bare ZnO, the spectra show that there is strong absorption in the ultraviolet region and a redshift. This could be due to the interaction of ZnO and NiO ions via sp-d exchange^50^. After adding NiO, there was a big shift in the way ZnO absorbed light toward the red end of the visible spectrum, which is good for photocatalytic and antibacterial activity by spreading light into the inner surface and making it reflect many times^26^. ZnO's excitation coefficient is directly linked to its absorption coefficient. When the absorption edges of ZnO nanoparticles get bigger, the optical band gap energy and the amount of light they can pass through go down^21^. In addition, the extrapolation of the hυ versus (αhυ)^2^ curve was used to determine the energy band gap for the prepared sample. The optical energy bandgap of the manufactured samples might be ascertained using some techniques, such as the Tauc relation. The formula for the Tauc relation is (αhυ)^n^ = A(hv E_g_), where hυ denotes the discrete energy bandgap of the light, α denotes the absorption, A denotes a constant that depends on the length of the localized state tails, and Eg denotes the optical energy bandgap. The band gap value was obtained by drawing a straight line on the curve and intersecting it with h at the X-axis shown in Fig. 5d^8^. When the NiO NPs are used as shells for ZnO NPs, the optical band gap energy falls from 3.19 eV (pure ZnO) to 2.966 eV. After adding the NiO (shell), the band gap shrank, indicating that the Ni^2^^+^ ions function as defect sites in the valence band to reduce the band gap. The quantum confinement effect can explain the decrease in bandgap energy and the increase in particle size. Grain boundaries play a significant role in the decline in optical band gap energy and transmittance, which leads to a higher density of grain boundaries and is advantageous for photocatalytic and antibacterial activity. The photocatalytic degradation percentage is quickly increased by the sonication process by increasing interaction and multiple reflection effects. This finding was in a good agreement with that of P. Gnanamozhi et al. and S. Al-Ariki et al.^21,26^.

### Contact angle

**Figure 6:**
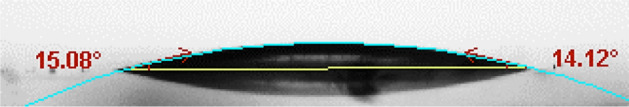
The CA of the prepared colloids (ZNO NPs).

The CA at the liquid–solid interface is used to assess the wettability of biomaterials in vitro. A low CA denotes a surface that is highly wettable or hydrophilic, which results in a continuous liquid film covering the solid surface. Three forces affect how wet a solid surface is: the surface tension of the solid, the surface tension of the liquid, and the interfacial tension^51,52^. Figure 6 shows the CA of the prepared ZNO NPs colloids, which seems to be between 15.08 and 14.12 which is an excellent result and it could be called a supper hydrophilic which indicates higher wettability. So, the colloids have low CA; therefore, the NP in the colloids has less interfacial tension which means there is no agglomeration in the particle, so it decreases the CA and increases the wettability on the surface. However, in the core shell, the attractive forces were lower among molecules with low CA, which decreased the tension of the surface^51^. Furthermore, this result agreed with TEM and FESEM results showing that low agglomeration in the particle due to the shell which decreases the attractive force between the core particles.

The CA results showed that the prepared ZNO NPs have high biocompatibility due to their excellent wettability, a crucial biomaterial property. Finally, higher wettability shows that colloids cover a larger area, which is a sign of strong antibacterial activity, and it is also suitable for a lot of biological applications.

### Antibacterial application

The antibacterial activity has been investigated using the well diffusion method on two types of bacteria, *E. coli* and *S. aureus*. Three diluted concentrations are used for ZNO NPs (25, 50, and 75 µg/mL) to indicate how the NP concentration will affect the bacteria (see Fig. S4). The inhibition zones of the prepaerd colloidal NP are listed in Table 1. The inhibition zone increased with the increase in NP concentration for both Gram-negative and Gram-positive bacteria. The antibacterial activity of the prepared NPs occurred because of the interaction between the NP surface and cell wall constituents; thus, structural changes may have been due to the membranes of the cells^53^.

**Table 1:** Inhibition zone using different concentrations of NiO@ZnO core–shell NPs.

Concentration µg/mL	Inhibition zone (mm)	
	*E. coli*	*S. aureus*
0.0	0.0	0.0
25	15.7	13.9
50	17.8	16.1
75	20.2	20.6

However, Gram-negative bacteria are composed of lipopolysaccharide in the outer membrane and a thin layer of peptidoglycan, which serves as a primary permeability barrier for macromolecules and hydrophobic drugs. On the contrary, Gram-positive bacteria have a simple structure with a membrane surrounding the cell as well as a cell wall composed primarily of a peptidoglycan layer and teichoic and lipoteichoic acids. Therefore, the inhibition zone in Gram-positive bacteria is larger than that in Gram-negative bacteria^54^. Damage to cell membranes was caused by direct contact with NPs. Given the removal of extra carboxylic groups from the cell surface, the bacterial cell as a whole had a negative charge at physiological pH^53,55^. Thus, the positive NPs become electrostatically bound to the negative cell surface, reducing cell activity. NPs’ penetration and toxicity killed and lysed the cells. Some studies have suggested that the small size of NPs may help them penetrate bacterial membranes. Outer cell membranes have nanometer-sized pores, and NPs can penetrate them^56,57^. Thus, membrane mass transfer is uncontrolled. NPs damage cell membranes via reactive oxygen species (ROS), such as superoxide (O^2−^) and hydroxyl (OH^−^) radicals, or direct cell damage. Metal oxide NPs produce superoxide and hydroxyl radicals, which damage cells. ROS oxidizes double bonds in phospholipids, increasing membrane fluidity and osmotic stress. ROS can damage iron-sulfur enzyme cofactors. NP size, concentration, and stability in the growth medium affect bactericidal activity^58,59^.

**Figure 7:**
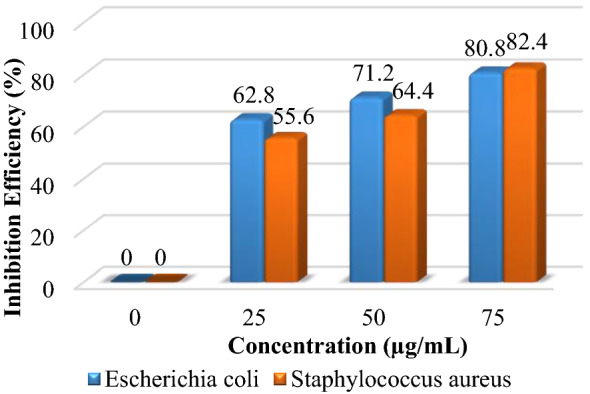
Inhibition efficiency of ZNO NPs.

The inhibition rates of ZNO NPs determined using Eq. (2) are shown in Fig. 7. The results showed a good inhibition rate for all concentrations, which were 62.8, 71.2, and 80.8 for *E. coli* and 55.6, 64.4, and 82.4 for *S. aureus*, respectively. These results are due to the tight contact of the NiO NPs (shell), which enabled the ZnO NPs (core) to contact tightly with the membrane and induce rupture of the contracted bacteria. The deposition or accumulation of the ZnO NPs on the surface of the bacteria, which leads to cytotoxic bacteria and a relatively increased ZnO concentration inside the cell, leads to cell death. Moreover, using the ZNO core–shell material will prevent the bacteria from nourishing themselves in the culture medium^55,60^. Furthermore, the NiO undergoes a transition through the cell's behavior, which aids the ZnO (core) in penetrating the bacterial barrier and dispersing the particle over a large area. Also, ZNO NPs may make it harder for the cell to make and break down heme components, which are needed for a number of heme-proteins to work properly and for the cell to get iron ions. These possibilities are demonstrated and proved with A. Kubacka et al. Study^61^. Therefore, core shelling the metal oxide with another metal oxide or other material improves its antibacterial activity to a higher level than using it alone. Comparing this result with another result like that of K. S. Khashan et al., our result exhibits a higher inhibition zone for prepared NPs^53^. Furthermore, when compared to the P. Gnanamozhi et al. study, which used Ni-doped ZnO NPs on Gram-negative and Gram-positive bacteria, the maximum inhibition on Gram-negative bacteria was 15 mm, while in our study it reached 20.2 mm^21^.

### Synthesized NPs induce the death of bacterial strains

**Figure 8:**
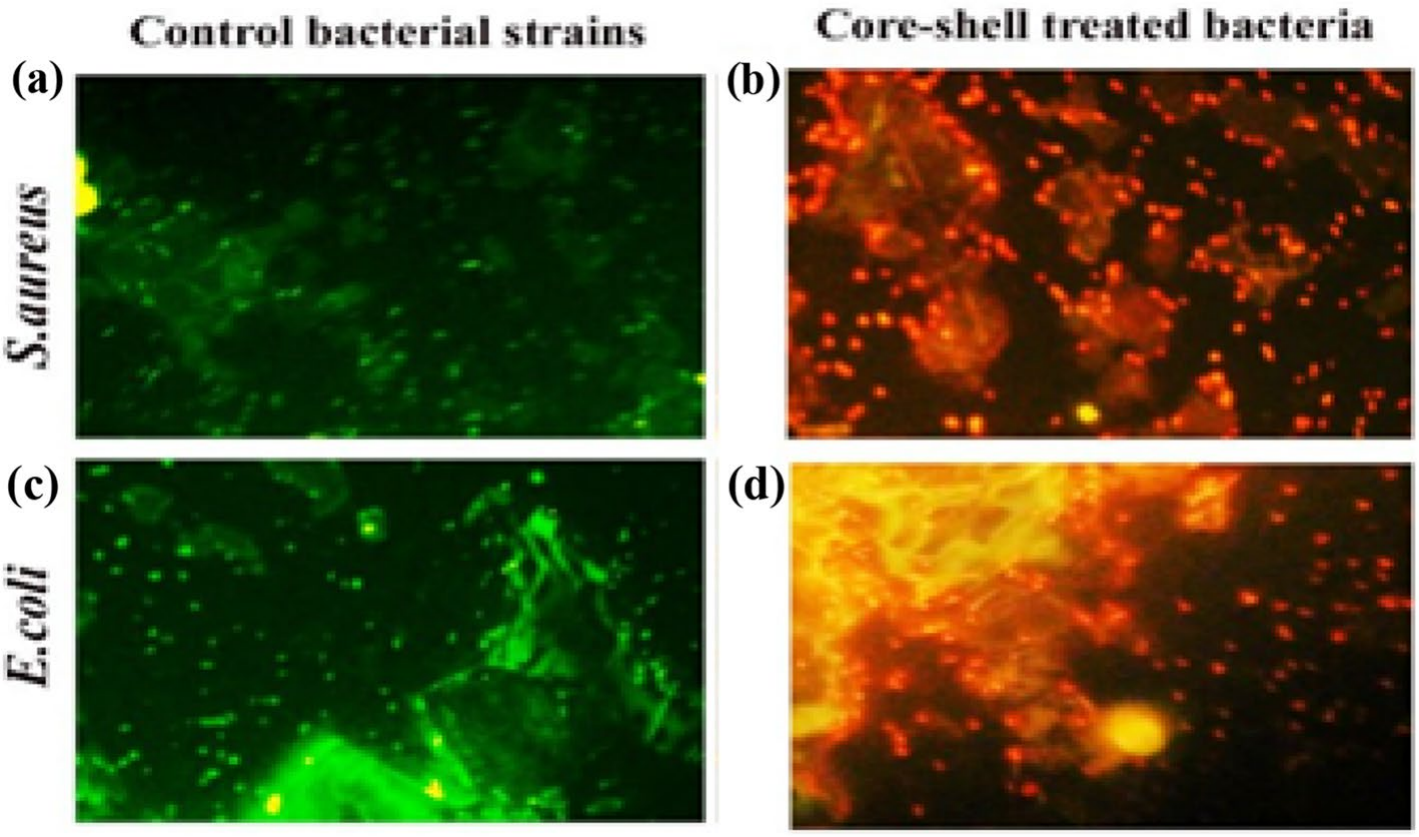
The fluorescence microscopic image of S.aureus and E.coli where (**a**) and (**c**) are the control bacterial strain respectively and (**b**), (**d**) are the treated bacteria with ZNO NPs.

**Figure 9:**
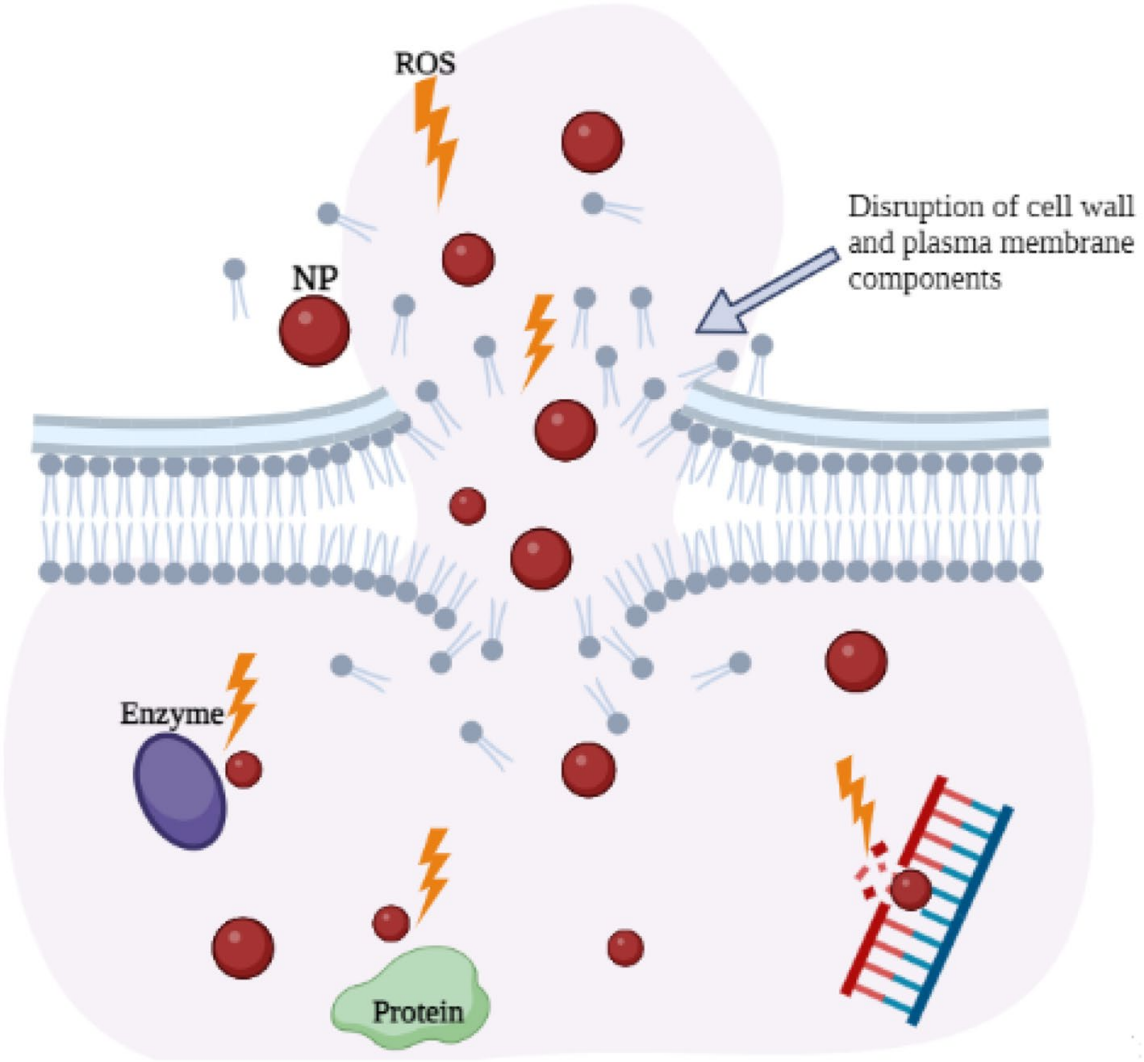
The illustration of killing mechanisms of nanoparticles against bacteria.

As shown in the Fig. 8, acridine orange/ethidium bromide (AO/EtBr) dual staining was used to determine which bacterial strains were alive and which were dead before fluorescence Microscopy was used to test the antibacterial activity of the produced nanoparticles on S. aureus and E. coli bacterial strains. When it binds to the nucleic acid of living bacteria, acridine orange emits a green fluorescence (Fig. 8a and c). On the other hand, ethidium bromide predominantly binds to the nucleic acid of dead bacteria and fluoresces red or orange (Fig. 7b and d)^62,63^. The color of living cells will therefore be green, whereas the color of dead cells will be red. All untreated bacterial cells for both types of bacterial strains fluoresced green in Fig. 8a and c, demonstrating their viability. Treatment with ZNO NPs causes almost all of the cells to become red, which shows that more cells are dead than in untreated cells. Therefore, ZNO NPs had the most effect on both kinds of bacteria^64^. Nanoparticles affected S. aureus more than E. coli due to variations in cell membrane structure. Nanomaterials can disrupt bacteria's DNA in a number of ways, including endogenous and exogenous harm. Reactive oxygen species (ROS) can form when endogenous sources attack. Normal cellular metabolism results in the production of ROS in cells^65^. These free radicals are incredibly unstable and can react with other substances in a split second. The free radicals and the DNA set off a series of events that led to the genotoxic lesions^66^. The killing mechanisms of nanoparticles against bacteria are illustrated in Fig. 9.

### Toxicity

**Figure 10:**
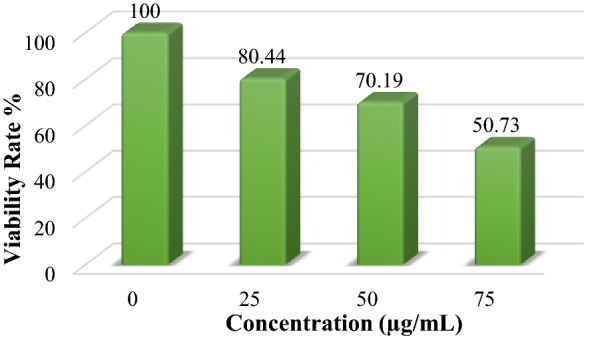
Cell viability rate of ZNO NPs.

The effect of the NPs on the cell viability of white blood cells has been measured using an ELISA microplate spectrophotometer. The activity of nanomaterials against the biological system and cells is a significant factor for biomaterial requirements, which makes them suitable for medical applications. Thus, the response of the white blood cells to ZNO core–shell NPs was investigated via an MTT assay after 24 h of exposure to NPs where the violet color of MTT becomes lighter with the increase in the percentage of nanomaterials, which means that the living cells have decreased (see Fig. S5). Figure 10 demonstrates the cell viability rate for the prepared NPs at different concentrations (0, 25, 50, and 75 µg/mL), which was determined using Eq. (3). The minimum cell viability at ZNO NPs concentration (75 g/mL) is 50.73%, which is still within the acceptable range.In addition, we can use the lower concentration to obtain the effect of ZNO NPs, which show good cell viability and lower cell toxicity. Moreover, the toxicity of ZNO NPs might be due to the high concentration of ZnO, whereas some studies indicate that increasing the ZnO concentration leads to more cell damage due to changes in the PH surrounding cell^55^.

## Conclusion

The proposed novel hybrid techniques successfully form the ZNO NPs core–shell.Based on the result, the prepared ZNO NPs have a small size, uniform distribution, and no aggregation because the NiO (shell) acts as an effective stabilizer and prevents the ZnO (core) from touching each other and coating them. The formation of the ZNO NPs core–shell is confirmed by XRD, FESEM, TEM, and EDX.The CA results showed that the prepared ZNO NPs have high biocompatibility due to their excellent wettability, a crucial biomaterial property. Also, higher wettability shows that colloids cover a larger area, which is a sign of strong antibacterial activity, and it is also suitable for a lot of biological applications. Also, the fluorescence microscopic image confirmed the bacterial death when using the ZNO NPs. The results show that the prepared ZNO NPs have excellent antibacterial activity against both Gram-negative and Gram-positive bacteria, even at low concentrations.Therefore, core shelling the metal oxide with another metal oxide or other material improves its antibacterial activity to a higher level than using it alone. Finally, the PLAL is an easily scalable, cost-effective, and environmentally friendly method for the synthesis of NPs, and the prepared core–shell NPs could be used in other biological applications such as drug delivery, cancer treatment, and further biomedical functionalization.

## Material and method

### Material

The Nickel and Zinc pallets are purchased from Sigma-Aldrich with a purity of 99.98% (the XRD result shown in Figs. S1, S2 for both pallets) with 1 mm thickness. The nickel with a dimension of 1 cm × 1 cm was used as a laser target and zinc with a 1 cm × 4 cm dimension was used as a plasma jet target. The distilled water, which was distilled using the Millipore water purification system, was used in this study.

### Instrument

The nickel and zinc targets are purified to remove the contamination from the surface using sanding (with a spatial paper) and a professional ultrasonic cleaner at the plasma lab, physics department, University of Baghdad, Iraq. Laser ablation was conducted using Q-switched Nd:YAG operated at a wavelength of 1064 nm. 800 mJ of laser energy with a 7 Hz repetition rate and a 10 ns pulse duration was employed for ablation. To perform laser ablation, the laser beam was focused on the target using a positive lens with a 9 cm focal length and a 2.5 mm spot size. The plasma jet contains argon gas (99% purity) and a locally made high-voltage power supply ranging between 10 and 20 kV. Atomic absorption spectrometry (AAS-Perkin Elmer, Analyst 400, 2014) was used to measure the concentration of NPs present in the colloidal solution, which measures the number of atoms in the part-per-billion range (ppm = µg/mL) at the University of Baghdad in the life sciences department. The AAS measurement was repeated three times for each sample, and the average was taken. X-ray diffraction (XRD) was used to examine the crystal structure of the produced ZnO@NiO (ZNO) core–shell NP using a kα-Cu target (λ = 0.15406 nm; the tube was operated at 45 kV, and the scan was acquired over a 2θ range of 20°–70°). The following Debye–Scherrer equation was used to calculate the grain size^16,67^:

1 $$ {\text{D}} = \left( {{\text{k }}\lambda } \right)/\left( {\beta {\text{ cos}}\alpha } \right), $$

where D is the average crystal size, β is the full width half maximum, k is the Scherrer constant (0.9), λ is the X-ray constant (0.15406 nm), and α is the Braggs angle^53^. The optical characteristics were measured using a UV–Vis spectrophotometer model (Metertech, SP8001 spectrophotometer, Japan) in the 100–1000 nm range at the University of Technology in the Laser and Optoelectronics Engineering Department. Field emission scan electron microscopy (FESEM) was used to evaluate the surface morphology, particle size, and chemical makeup of the produced NPs utilizing a JSM-IT800 (origin) equipped with energy-dispersive X-ray technology (EDX) in DayPetroinc Co. Ltd. Tehran, Iran. Transmission electron microscopy (TEM, Zeiss, Germany) is used at DayPetroinc Co. Ltd. in Tehran, Iran to study the size and shape of the particles as well as the growth of the core–shell.

### Nanoparticle synthesis

The ZNO core@shell was synthesized using hybrid techniques where the nickel target was ablated in distilled water using PLAL techniques for 10 min at room temperature, then the produced colloidal, which contains NiO NPs, was used and the titanium target was immersed in it. The zinc target was ablated using the plasma jet technique for 10 min and a 3 L/min gas follow rat.

### Antibacterial

Two clinical isolates, Escherichia coli (gram-negative) and Staphylococcus aureus (gram-positive), were used to evaluate the antibacterial activity of ZNO NPs. These two bacterial isolates were transferred to Mueller–Hinton agar medium, incubated overnight at 37 °C, and kept in the refrigerator at 4 °C until required. Micropipette tips were used to create wells with diameters of roughly 6 mm on the surface of agar media, which were then filled with NP suspensions of varying concentrations. These plates were incubated for 24 h. The antibacterial efficacy of ZNO NPs was determined by measuring the widths of inhibition zones from many directions using a ruler. All experiments were conducted in duplicate, and purified water served as the negative control^68^. The antimicrobial activity percentage (%) was calculated using the following formula^18^:

2 $$ {\text{Activity}}\,{\text{ Index}} = \frac{{{\text{ The }}\,{\text{Inhibition }}\,{\text{Zone }}\,{\text{tested }}\,{\text{the}}\,{\text{ sample}}\,{ }\left( {{\text{diameter}}} \right)}}{{{\text{ The}}\,{\text{ Inhibition }}\,{\text{Zone }}\,{\text{tested }}\,{\text{by }}\,{\text{the}}\,{\text{standard }}\,\left( {{\text{diameter}}} \right){ }}}*{1}00. $$

The standard used in this work is 250 µg of Emoxilian antibiotic with a 25 mm inhibition zone.

### Fluorescent Microscopic Imaging

To determine whether bacterial strains were alive or dead based on membrane integrity, fluorescent microscopic imaging was used to examine the antibacterial activity of as-prepared NPs on *E. coli* and *S. aureus* bacteria. In contrast to ethidium bromide (EB), which fluoresces red when bacteria are dead, acridine orange (AO), which fluoresces green, was used to stain live bacterial strains. In a nutshell, *E. coli* and *S. aureus* bacteria were put in Eppendorf before and after being treated with NPs. After that, 10 g/ml of AO/EB was added, and the mixture was left alone for 2 min. The samples were immediately examined under fluorescent microscopy prior to bacteria leaking their dyes.

### Availability

The cell viability test was investigated using human white blood cells (the blood was collected from healthy volunteers aged 25 years) under highly sterile conditions. White blood cells with a density of 1*10^4^ cells/mL in a well plate were exposed to NPs and then incubated at 37 °C for 24 h. Thereafter, the plate was poured out and washed with PBS three times to remove any trace of NPs. Then, 10 μL of MTT dye solution was added to the well plates and incubated for 4 h at 37 °C. The cells were washed several times with PBS until the excess dye was removed. After the plates were completely dry, the results were read using an ELISA microplate spectrophotometer at a wavelength of 500 nm. The percentage of living cells relative to the total number of cells was used to calculate cell viability; this percentage was calculated using the following equation^18^:

3 $$ {\text{Viability }}\left( {\text{\% }} \right) = \frac{{{\text{Mean }}\,{\text{optical }}\,{\text{density}}\,{\text{ of}}\,{\text{ test }}\,{\text{samples}}}}{{{\text{Mean }}\,{\text{optical }}\,{\text{density}}\,{\text{ of }}\,{\text{the }}\,{\text{control }}}}*{1}00. $$

All procedures were performed according to guideline numbered (UOT-LOEE-17082020) approved by the human Care and Ethics Committee at Biotechnology Division, Laser and Optoelectronics Engineering Department, University of Technology, Baghdad, Iraq.

### Blood collation and preparation

Fresh samples of blood were taken from healthy donors and distributed into heparin-coated tubes based on the method of the National Institute of Health and the Food and Drug Administration and as per the declaration and regulation of Helsinki of 1975 as a statement of ethical principles. Permission was obtained from the hospitals of the medical city in Baghdad, Iraq, and approved by the institutional ethical committee of the University of Technology, Baghdad, Iraq (Ref. No. LOEE 13–17–08–2020). The study participants were informed about the value of the study before we began to collect any data or samples. Informed consent and/or assent were obtained from the study participants.

## Supplementary Information

Supplementary Information.

## Data Availability

The datasets used and/or analysed during the current study available from the corresponding author on reasonable request.
