# Supplementary material for: A novel method for ZnO@NiO core–shell nanoparticle synthesis using pulse laser ablation in liquid and plasma jet techniques
Source: Sci Rep. 2023 Apr 3;13:5441. doi: 10.1038/s41598-023-32330-z (PMC10070463; doi:10.1038/s41598-023-32330-z)
Supplement: Supplementary file 1 — Supplementary Information. [file 41598_2023_32330_MOESM1_ESM.docx]

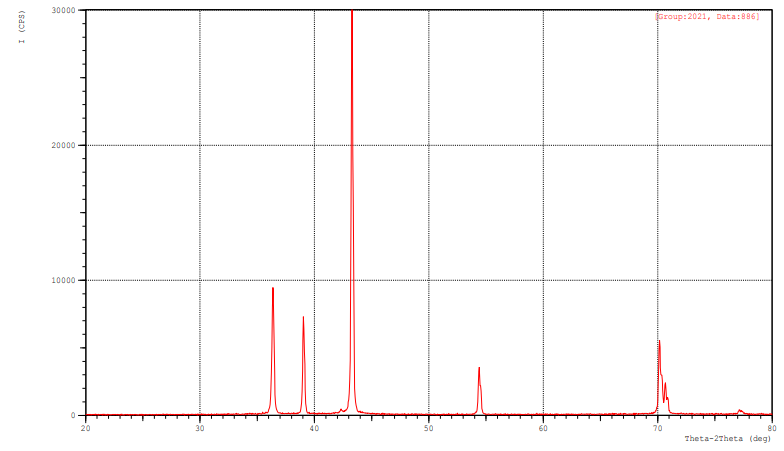


**Figure S1:** The XRD of a zinc pallet, showing the main peaks of Zn and the purity of the used pallet.


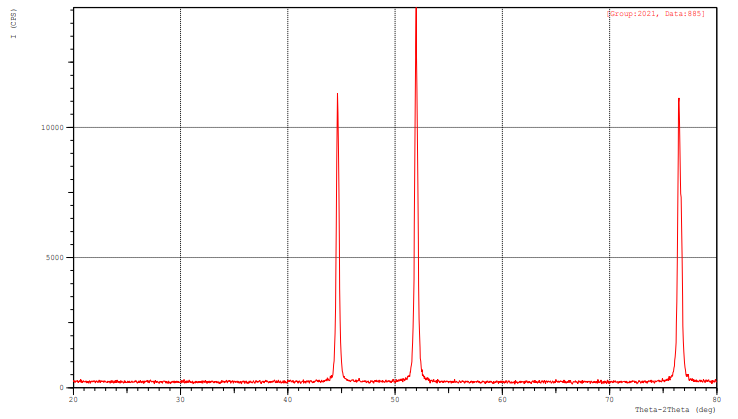


**Figure S2:** The XRD of a nickel pallet, showing the main peaks of Ni and the purity of the used pallet.


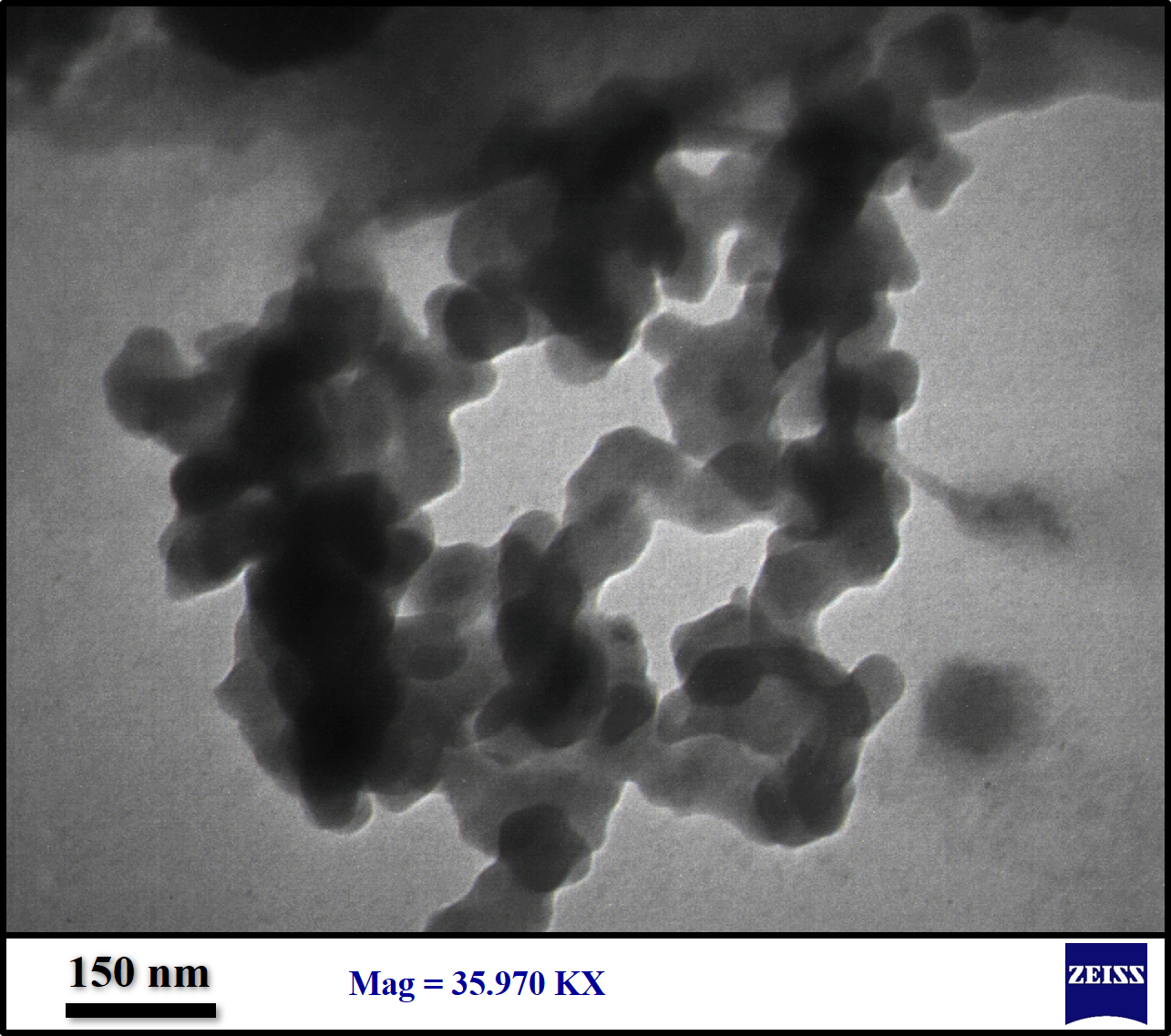


**Figure S3:** the TEM image for NiO before made the core/shell.

| **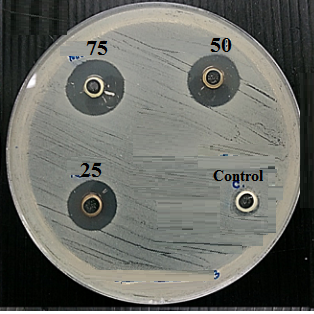(a)** | **(b)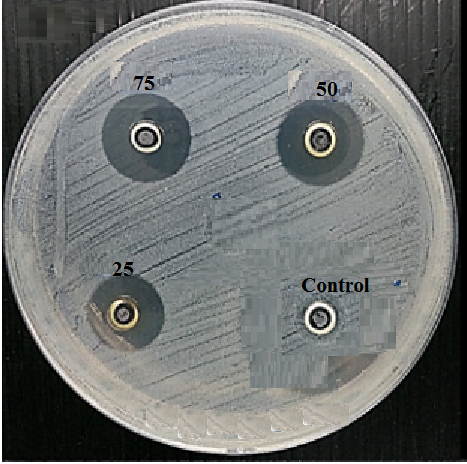** |
| --- | --- |

**Figure S4:** the inhibition zone of the used bacteria at (25, 50, and 75 µg/mL) concentration of the prepared ZNO NPs, (a) *Escherichia coli,* (b) *Staphylococcus aureus.*

**Table S1:** the XRD peaks details of ZNO NPs.

| **Element** | ***2*θ(deg)** | **β=FWHM(deg)** | **D (nm)=0.9λ/βcosθ** | **Average (nm)** | ***h*** | ***k*** | ***l*** |
| --- | --- | --- | --- | --- | --- | --- | --- |
| ZnO | 31.856 | 0.6581 | 12.56966663 | 13.81 | 1 | 0 | 0 |
|  | 34.52 | 0.575 | 14.48627299 |  | 0 | 0 | 2 |
|  | 36.346 | 0.6522 | 12.83673426 |  | 1 | 0 | 1 |
|  | 47.616 | 0.8479 | 10.25400612 |  | 0 | 1 | 2 |
|  | 56.652 | 0.7119 | 12.69352952 |  | 1 | 1 | 0 |
|  | 66.412 | 0.5465 | 17.39601829 |  | 2 | 0 | 0 |
|  | 68.035 | 0.6613 | 14.51209702 |  | 1 | 1 | 2 |
|  | 69.221 | 0.6134 | 15.7562463 |  | 2 | 0 | 1 |
| NiO | 62.945 | 0.7033 | 13.26109348 | 11.08 | 1 | 1 | 1 |
|  | 75.435 | 1.2 | 8.379836017 |  | 2 | 0 | 0 |
|  | 79.621 | 0.9 | 11.50577036 |  | 2 | 2 | 0 |

**
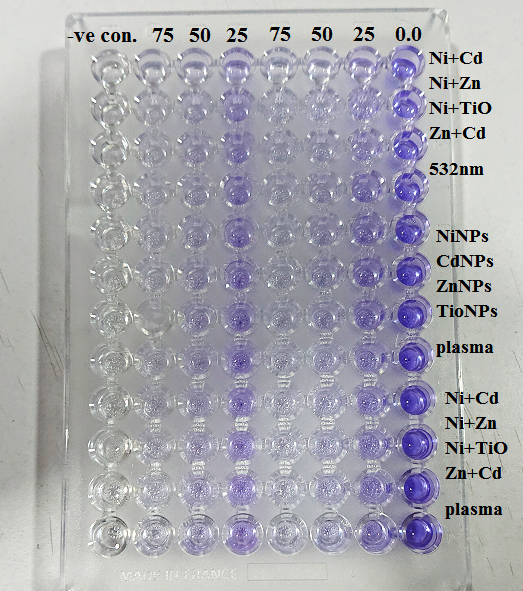
**

-cv 75% 50% 25% 75% 50% 25% 0%

**Figure S5:** MTT assay after 24 h of exposure to NPs (for all used concentration 0, 25, 50, 75% and the negative control (-cv)) where the violet color of MTT becomes lighter with the increase in the percentage of nanomaterials, which means that the living cells have decreased. For each used concentration the MTT assay was repeated four times.
